# Surface neatness as an index of aesthetic value of everyday objects

**DOI:** 10.3389/fpsyg.2025.1578785

**Published:** 2025-07-10

**Authors:** Tatiana Ledneva, Andriy Myachykov, Yury Shtyrov

**Affiliations:** ^1^Institute for Cognitive Neuroscience, HSE University, Moscow, Russia; ^2^Center for Cognitive and Brain Sciences, University of Macau, Taipa, China; ^3^Center of Functionally Integrative Neuroscience, Aarhus University, Aarhus, Denmark

**Keywords:** surface perception, visual attractiveness, aesthetics, human-object interaction, neatness, affordances

## Abstract

**Introduction:**

Surface neatness is a fundamental yet underexplored determinant of the aesthetic evaluation of everyday objects. While prior research has typically examined individual surface features - such as gloss, shine, dirt, or scratches - in isolation, the holistic impact of surface neatness has received little systematic attention.

**Methods:**

In this study, participants viewed images of objects from five categories (household items, tools, personal use items, stationery, and kitchen utensils), each presented in three surface conditions: untidy (displaying mechanical and hygienic defects), neutral (without visible defects), and neat (exhibiting gloss and cleanliness). For each object, participants provided a preference rating reflecting their aesthetic evaluation.

**Results:**

Analysis revealed a robust effect of surface neatness on aesthetic preference: objects in the untidy condition consistently received the lowest ratings, while neat surfaces were rated most attractive. The differences between all surface conditions were statistically significant.

**Discussion:**

These results demonstrate that surface neatness is a dynamic and salient factor shaping the perceived value and desirability of everyday objects. The findings underscore the need for more rigorous operationalization of surface properties in empirical research on human-object interaction and suggest practical applications for product design, consumer psychology, and sustainable practices, where surface conditions directly influence aesthetic experience and object appeal.

## Introduction

How we perceive an object’s surface plays a central role in how we recognize, evaluate, and interact with it, both visually and behaviorally. In cognitive psychology, surface properties such as gloss, color, and texture influence attention allocation, object categorization, and motor affordance activation (Fleming, 2014; Myachykov et al., 2014; Neumann et al., 2007; Tucker and Ellis, 1998). These perceptual cues can modulate an object’s salience during visual search (Wolfe and Horowitz, 2017), shape expectations of usability or contamination (Curtis et al., 2011), and determine interaction likelihood (Creem and Proffitt, 2001). Thus, surface features are not merely decorative, but rather functionally diagnostic as they provide information about an object’s condition and potential for action. This view is consistent with ecological and embodied theories of perception (Xenakis and Arnellos, 2013).

Recent work in empirical aesthetics suggests that visual cues, such as those described above, contribute to users’ experiences by influencing judgments of appeal, harmony, and desirability (Leder and Nadal, 2014; Palmer et al., 2012). However, despite the acknowledged importance of surface cues, most existing studies focus on isolated properties, such as gloss or texture, without considering them as part of a broader framework of surface appearance as a continuum. This fragmentation limits our understanding of how multiple surface indicators collectively shape aesthetic judgments and behavioral tendencies in real-world settings. Importantly, examining these isolated properties is insufficient for our understanding of the diverse aesthetic experiences related to object perception and use in contexts beyond laboratory settings. In real-world settings, surfaces constantly evolve due to both human interaction and environmental factors (Karana et al., 2017). These changes include signs of wear, scratches, stains, decorations and other alterations in surface neatness. Although existing research emphasizes the significance of *surface neatness* in the perception of everyday objects (Augustin et al., 2012; Leddy, 1995, 1997; Saito, 2001), and the valency of some visual cues of neatness has been examined across different contexts (Chadwick and Kentridge, 2015; Cheng et al., 2022; Collis et al., 2023; Di Muro and Noseworthy, 2013; Ostuzzi et al., 2011; Powell, 2021; Zhong et al., 2010), there is a paucity of systematic research on the relationship between surface neatness and aesthetic evaluation. The current study, therefore, aims to investigate how changes in surface neatness within a single object influence its aesthetic value, thereby endeavoring to contribute to a deeper understanding of the role of surface properties in aesthetic perception.

In empirical aesthetics, aesthetic value is frequently operationalized through subjective judgments of visual preference, such as how much an observer likes or dislikes a stimulus (Jacobsen et al., 2006; Leder and Nadal, 2014). In the present study, we also adopt this approach and interpret preference as a proxy for aesthetic value. While this type of rating may not capture the full complexity of aesthetic experience, it is widely accepted as an ecologically valid and theoretically grounded measure of perceived visual appeal, especially in studies involving familiar everyday objects (Palmer et al., 2012; Vessel et al., 2014).

‘Surface neatness’ can be defined as the visually perceived state of cleanliness or contamination of an object (Merriam-Webster, n.d.). In the field of everyday aesthetics—a branch of philosophy dedicated to the study of aesthetic experiences and values in daily life—neatness is recognized as a significant aesthetic property of objects (Leddy, 1995, 1997; Saito, 2001). The concept of neatness is frequently associated with cultural and social norms that influence perceptions of cleanliness and order. To illustrate, Leddy (1995) proposes that the perception of neatness is inextricably linked to primary aesthetic responses that are formed during early childhood, as individuals learn to distinguish and appreciate cleanliness and order in their surroundings.

It is important to note that the concept of neatness can also be understood in a broader sense, encompassing its more general and metaphoric significance. As Leddy (1995) emphasizes, neatness can be considered a proto-aesthetic property, evolving from the hygienic evaluation of surfaces to its metaphorical connotations. This is evident in expressions such as “spotless reputation” and “messy thoughts,” highlighting its relevance beyond the physical realm. In this study, however, we adopt a deliberately narrow interpretation of neatness, defining it as the visible traces of the object’s use, including dirt, stains, scratches, or wear. This operational definition reduces subjectivity, also enabling the standardization of stimuli. Although our operational definition is necessarily narrowly focused on visible signs of tear and wear, such as dirt, stains, and scratches, we acknowledge that perceived neatness in real-world settings may include the absence of defects and the presence of positive surface cues, such as gloss, brightness, and subtle visual order. This perspective corroborates findings that clean, shiny surfaces are often perceived as neat because they lack visible signs of use and appear well-maintained (Leddy, 1997). Thus, our manipulation included both negative (untidiness) and positive (enhanced neatness) deviations from a neutral baseline.

Surface neatness can be evaluated via specific visual cues, e.g., presence of gloss, shine, and surface cleanliness (Leddy, 1997). Gloss and shine are also associated with a surface’s ability to reflect light, enhancing perceptions of a pristine and well-maintained state (Chadwick and Kentridge, 2015). In contrast, dirt, stains, and visible signs of use and wear contribute to the perception of untidiness (Baxter et al., 2016; Leddy, 1995). Baxter et al. (2016) define a “used” object as one that has left its original, new condition, with “wear” referring to the physical outcome of usage. “Indicators of use” include signs of wear but can also encompass contextual factors that signal use indirectly/implicitly.

The presence of traces of use on the surface conveys a message to the observer regarding the possibilities for interaction with the object, its potential and the likelihood of successful engagement. These characteristics are particularly significant in the context of studying sensorimotor effects. For instance, descriptions of an object’s surface state can influence an observer’s behavior, either accelerating or delaying interaction (Buccino et al., 2009; Garofalo et al., 2021; Mugge et al., 2018). Furthermore, research by Garofalo et al. (2021) shows that information signaling pleasant interaction with the object’s surface (e.g., smooth) speeds up reach-to-grasp task performance, while negative attributes (e.g., sticky) have the opposite effect. However, these findings are primarily based on linguistic stimuli rather than visual ones. In contrast, our study focuses on the visual characteristics of surfaces, such as cleanliness and dirtiness, interpreting them as a “history” of the object’s use. These considerations are especially important when studying everyday objects and tools, as they are designed for regular interaction and must provide the user with the information about their functionality and the ease of use. This perspective aligns with that proposed by Xenakis and Arnellos (2013), who argue that aesthetic perception arises from the evaluation of interactive affordances and emphasize that aesthetics is not static but rather dynamic and context-dependent, playing a critical role in anticipating and assessing the success of interaction with the object.

Most existing studies agree that neatness is linked to positive valence, while untidiness is associated with negative valence. Features that define surface neatness, such as gloss, shine, or the absence of contaminants, have been shown in various studies to significantly influence consumer preferences and enhance perceived object’s value (Sharma and Kumar, 2023; Silvia et al., 2018). For example, Silvia et al. (2018) found that participants rated shiny, mirror-polished silver coins and copper cylinders as more attractive and of higher quality compared to their matte counterparts. The authors offered an evolutionary explanation: Glossy surfaces may instinctively appeal to humans as they resemble clean water, a vital resource for survival. Further, Sharma and Kumar (2023) demonstrated that gloss and reflections enhance perceived product attractiveness in advertisements. However, this effect was attenuated for complex product designs, where visual complexity diverted attention from the details of the surface.

At the same time, surface imperfections, including dirt, stains, scratches, and dents, exert a considerable influence on consumer preferences and diminish the perceived value and attractiveness of an object. One of the earliest studies on the topic by O’Reilly et al. (1984) demonstrated that contamination can have a significant impact on purchasing intentions prompting the authors to identify neatness as a pivotal factor in consumer decision-making, particularly in the context of purchasing used goods. Surface imperfections, such as dirt or wear, often evoke negative responses, including concerns about hygiene, which can act as psychological barriers to purchase. Furthermore, Di Muro and Noseworthy (2013) demonstrated that the physical condition of money, particularly its degree of wear, has a significant impact on consumer behavior. Namely, the habit of spending worn banknotes while retaining newer, crisper ones may be driven by the feeling of disgust. That is, the association between signs of use and potential contamination is likely to elicit a disgust response, which in turn motivates individuals to dispose of untidy items more readily. Moreover, the relationship between surface neatness and product perception also extends to product packaging (White et al., 2016). This study showed that even minor surface imperfections, such as dents, scratches, and scuffs on packaging, are perceived as contamination cues. These imperfections resulted in adverse consumer reactions, despite the absence of actual risk to product quality or safety. The findings emphasize that neatness, or the absence of visible flaws, is of paramount importance in influencing consumer perceptions, as even minor defects can evoke automatic associations with contamination, thereby reducing the product’s attractiveness and desirability.

The studies reviewed above clearly show that specific visual cues related to surface neatness play a crucial role in shaping perceived value and aesthetic judgments of objects. However, a significant shortcoming of many of these studies is their somewhat fragmented approach, often focused on examining discrete elements of surface conditions in isolation, rather than considering them as part of a comprehensive continuum of surface neatness, with majority of research tacking single features, typically contrasting a neutral state with a modified state with either negative (e.g., scratches or stains; Mugge et al., 2018; Ostuzzi et al., 2011; White et al., 2016) or positive (e.g., added gloss or shine; Sharma and Kumar, 2023; Silvia et al., 2018) alterations. One noteworthy exception is the study by Di Muro and Noseworthy (2013), which examined the perception of copper coins by comparing two valence surface conditions: shiny versus matte or aged surfaces. However, this study did not include a neutral surface condition, and, furthermore, focused on a highly specific object class—money—whose multifaceted value complicates the generalizability of the findings.

To the best of our knowledge, no study to date has adopted a systematic approach to investigating how dynamic alterations in surface neatness influence the perception of an object’s aesthetic value. This gap in the literature highlights the need for a more integrated methodology to understand how the aesthetic value of objects evolves in response to real-world interactions, where surface conditions are subject to continuous change due to both use and environmental factors (Lilley et al., 2019). Here, we propose that surface imperfections, such as dirt, stains, scratches, and dents, and enhancements, such as gloss, shine, and cleanliness, can be viewed as a continuum of surface neatness. This approach is particularly relevant considering the nature of everyday objects (Augustin et al., 2012; Stich et al., 2007). In contrast to art, human faces and natural phenomena, everyday objects are intended for practical use and undergo constant change, which affects user interaction and satisfaction (Karana et al., 2016; Woolley, 2003). This dynamic is frequently conceptualized through the lens of the object’s lifecycle, which illustrates that, through use, objects undergo both reversible changes (e.g., contamination) and irreversible alterations (e.g., wear and damage) (Lilley et al., 2019; Ostuzzi et al., 2011; Woolley, 2003). An object’s surface is therefore inherently dynamic. Accordingly, we put forth an approach grounded in the notion of surface neatness, which regards it as a continuum encompassing both positive and negative modifications within a dynamic, real-world context.

In order to investigate how variations in surface neatness influence participants’ visual preference judgments in a systematic manner, we conducted a study, in which the participants were asked to rate how much they liked or disliked the appearance of everyday objects, presented under varying surface conditions. A set of realistic visual stimuli was created, depicting objects from five categories: household items, stationery, kitchen utensils, tools, and personal use items. Each object within these categories was presented in three distinct surface conditions: an untidy condition, featuring mechanical and hygienic imperfections such as scratches, dirt, and wear; a neutral condition, lacking visible flaws or decorative elements; and a neat condition, exhibiting signs of cleanliness, shine, or gloss. This design enabled us to simulate the dynamic nature of surface conditions, reflecting different stages of an object’s lifecycle as influenced by human use. The neat condition represents a brand-new or restored state, the neutral condition represents objects without noticeable defects, and the untidy condition reflects a clearly used and worn state. By employing a diverse array of 42 everyday objects from a range of categories as stimuli, our study aims to enhance the generalizability of the findings in comparison to prior research that frequently relies on a more limited range of objects.

Furthermore, we included object category as the second factor in our design. This decision was motivated by growing evidence that aesthetic preferences are often modified by the type of object to which a purported visual aesthetic property is applied. For instance, studies have demonstrated that the perceived attractiveness of color (Palmer and Schloss, 2010; Schloss et al., 2013), gloss (Spence, 2021; Ye et al., 2020), symmetry (Bertamini et al., 2019), and typicality (Halberstadt et al., 2003) varies substantially depending on the object type. While gloss is typically associated with premium quality, it may reduce aesthetic appeal in food-related contexts due to associations with artificiality or unhealthiness (Ye et al., 2020). Similarly, color preferences often reflect congruence between object function and surface appearance (Schloss et al., 2013). Based on these findings, we hypothesized that the effect of surface neatness would differ across categories. For instance, objects associated with physical contact or hygiene (e.g., kitchen utensils and personal items) may elicit a stronger negative reaction when untidy than tools or household equipment would. So, including object category as a factor allowed us to test not only the generalizability of neatness effects but also their potential interaction with the functional and normative expectations embedded in different object types.

As explained in more detail below, the stimuli in the present study were comprised of static images; nevertheless, we conceptualize surface neatness as a dynamic property. This is not in terms of temporal presentation, but rather as a representation of the distinct phases in an object’s real-world wear-and-clean cycle. This includes transitions from deterioration induced by use (e.g., dirt and scratches) to neutral maintenance to deliberate enhancement (e.g., polishing and restoration). Indeed, previous studies have shown that aesthetic value and perceived usability evolve over time due to physical and visual changes to an object’s surface (Karana et al., 2016; Lilley et al., 2019; Mugge et al., 2018). Our three-condition design emulates this continuum: the untidy state simulates a visibly worn or contaminated object; the neutral state depicts a maintained but unadorned object; and the neat state reflects surface improvement or active care. Thus, the “dynamic” quality of surface neatness refers to the simulation of temporally situated surface conditions that mirror real-world object use and maintenance cycles.

We hypothesized (H1) that variations in surface neatness would significantly affect participants’ subjective ratings of visual preference. Specifically, we predicted that objects with mechanical and hygienic imperfections (untidy) would receive lower ratings than those in the neutral condition. We also predicted that objects displaying neatness cues, such as gloss and shine, would receive the highest ratings. Furthermore, based on evidence that aesthetic judgments are modulated by object type, we hypothesized (H2) that the impact of surface neatness would differ across object categories. Specifically, we anticipated that objects associated with direct bodily contact or food preparation/consumption (e.g., kitchen utensils and personal items) would elicit a stronger negative preference response to untidiness than categories such as tools or household equipment.

## Methods and materials

### Sample size estimation

Ad-hoc power analysis was conducted using PANGEA v0.2 software (Westfall, 2015; https://jakewestfall.shinyapps.io/pangea). Given the lack of comparable studies, it was not possible to predict in advance the effect sizes and variances associated with the interaction. Accordingly, the default variance parameters in PANGEA (var[error] = 0.333, var[participant*surface*category] = 0.083) were employed to estimate the power for the interaction with a 3 (Surface State: Untidy, Neutral, Neat) × 5 (Object Category: Household Items, Tools, Personal Use Items, Stationery, and Kitchen Utensils) design. The results indicated that a sample size of 50 participants would be sufficient to detect an interaction between surface state and object category with a medium effect size (*d* = 0.45) at a power of 99.9%.

### Participants

Fifty participants (24 females, 26 males) with an average age of 24.5 years (SD = 9.1) took part in the study. All participants were native Russian speakers with normal or corrected-to-normal vision and no history of neurological or psychiatric disorders. No participants had received formal training in fine arts, design, or architecture. To control for the possibility that stimuli displaying signs of contamination might evoke an excessive negative response in some particularly sensitive individuals, potentially due to associations with feelings of disgust (Rozin and Fallon, 1987), the Russian version of the Maudsley Obsessive-Compulsive Inventory (MOCI; Hodgson and Rachman, 1977; Karpov et al., 2022) was included as part of the mandatory questionnaire for all participants, as OCD is known to be associated with negative/disgust bias (Knowles et al., 2018). The mean score on the MOCI was 7.88 (SD = 3.15, range 2–16). All participants provided written informed consent, received compensation for their time, and were informed of their right to withdraw from the study at any time.

### Materials

The stimulus set consisted of 126 images, including 42 original photographs of everyday objects from the well-established Bank of Standardized Stimuli (BOSS) database (Brodeur et al., 2014) and their modified (see below) versions. All selected objects were intended for manual manipulation and were grouped into five categories: household items (e.g., scrub brush, clothespin), tools (e.g., hammer, chisel), personal use items (e.g., toothbrush, comb), stationery (e.g., stapler, pencil), and kitchen utensils (e.g., fork, ladle). These five categories were selected via a principled selection process from the 21 object categories available in the BOSS database. We excluded categories that lacked a manipulable surface (e.g., nature scenes), were likely to evoke strong cultural or stylistic associations (e.g., fashion accessories, electronics, toys, jewelry, vintage cars), or contained food-related content where surface cleanliness is confounded with freshness or edibility.

The remaining categories included objects that are commonly encountered in everyday settings and do not carry strong cultural, historical, or stylistic connotations. Importantly, these types of objects have been widely used in previous research on affordances and manual interaction (Borghi and Riggio, 2009; Myachykov et al., 2013; Tucker and Ellis, 1998, 2004). This allowed us to focus on stimuli that were not only representative of everyday object use, but also empirically grounded in the cognitive and perceptual literature, thereby ensuring both ecological validity and methodological rigor.

In order to operationalize the neat and untidy surface states of everyday objects, we applied subtle modifications to these original images without changing their overall content. These modifications were based on previous findings, which identified key visual attributes associated with surface neatness (Leddy, 1995, 1997): the untidy surface state is characterized by the presence of hygienic or mechanical imperfections, including dirt, stains, signs of use, scratches, scuffs, and similar defects, whereas, in contrast, the neat surface state involves the incorporation of such characteristics as shine, gloss, or simple decorative elements.

An image-editing protocol was developed to control for manipulations of the objects’ surface characteristics. This protocol was based on a predefined set of visual criteria and operational rules for each experimental condition. A professional designer performed all image manipulations using Adobe Photoshop 2022. The process adhered to a formalized procedure that had been reviewed and approved by the project’s art director (Supplementary material 1). The primary objective was to simulate three distinct surface states—neat, neutral, and untidy—while preserving the identity and visual recognizability of each object and minimizing potential semantic and cultural biases.

#### Neutral condition

The original, unmodified photographs sourced directly from the BOSS database served as the stimuli in this condition. These images depicted objects in a usage-neutral state, specifically devoid of added gloss, visible contamination, or stylistic embellishments.

#### Untidy condition

Visual alterations were implemented to simulate mechanical and hygienic imperfections. The imperfections were chosen from a predefined list of surface defects commonly associated with wear and contamination. Specific modifications included:

1. Scratches rendered as linear disruptions with low opacity and eroded edges.
2. Smudges and grease marks applied as semi-transparent overlays with variable translucency, typically ranging from 30 to 70%.
3. Stains, fingerprints, and dust particles incorporated to enhance the perception of dirt and use.

#### Neat condition

Modifications were made to improve the appearance and perceived level of cleanliness. While the untidy condition directly reflected surface degradation in the form of dirt, stains, and scuffs, the neat condition was designed to represent a distinctly maintained and visually enhanced surface state. Rather than merely omitting imperfections, we introduced positive visual indicators of surface care—including gloss effects, shine gradients, and subtle geometric elements—which have been shown to increase perceived cleanliness and visual order. This manipulation reflects an expanded, ecologically grounded understanding of neatness, supported by literature on everyday aesthetics (Leddy, 1997), whereby polish, reflectivity, and subtle detailing are interpreted as signs of attention, upkeep, and desirability. Therefore, our operationalization of neatness is best conceptualized not as a binary (clean vs. dirty), but as a perceptual continuum ranging from degradation to enhancement. The alterations included:

1. Gloss effects were created through simulated highlight overlays and are characterized by soft edges and high luminance contrast. They mimic polished surfaces.
2. Shine gradients employed to further enhance the perception of a well-maintained, reflective surface.
3. Optional decorative patterns (e.g., subtle geometric motifs) were added to approximately 20% of the objects. Importantly, this balances the untidy stimuli in terms of perceived complexity. The patterns were strictly constrained to avoid conveying symbolic meaning, excessive color saturation, or specific stylistic cues.

**Figure 1:**
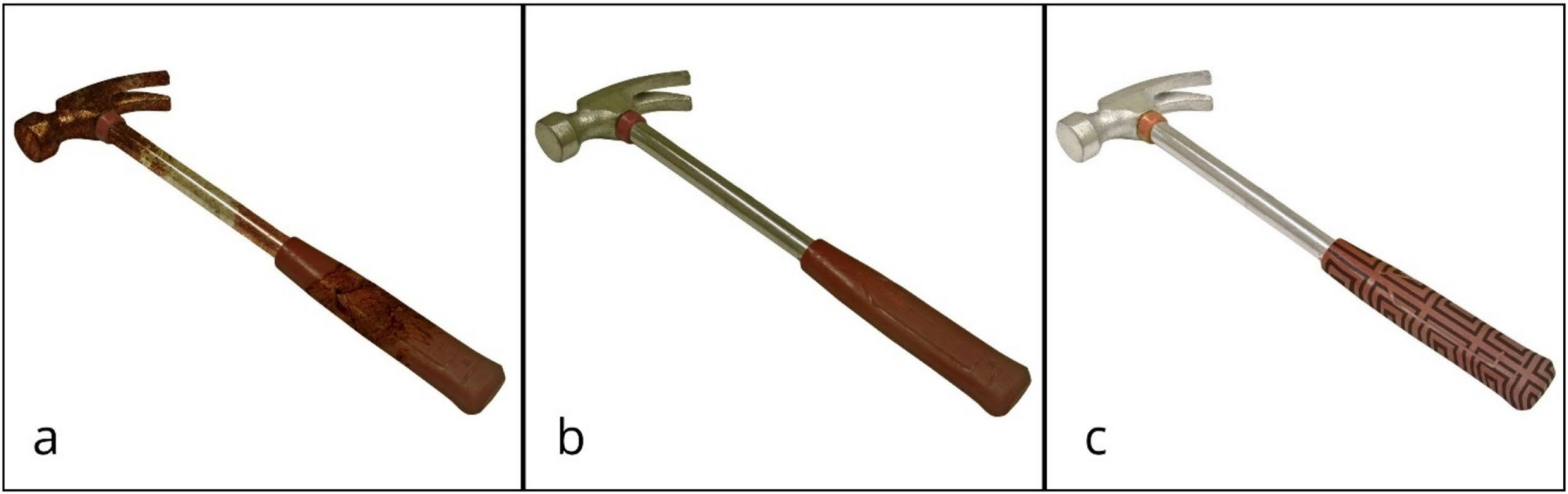
Examples of stimuli used in three experimental conditions: **(a)** untidy, **(b)** neutral, and **(c)** neat. Base images were sourced from the BOSS database (Brodeur et al., 2014), modified to alter surface neatness, and are used here under the Creative Commons Attribution-ShareAlike 4.0 International License (https://creativecommons.org/licenses/by-sa/4.0/).

All manipulations were implemented using Adobe Photoshop 2022. The stimuli were rendered in PNG format with dimensions of 2,000 × 2,000 pixels and a 32-bit color and centered on a square canvas (see examples in Figure 1).

To ensure rigorous control and standardization of quality across the entire stimulus set, a single professional designer performed all image edits. The project’s art director continuously oversaw this process and conducted a comprehensive review of every modified stimulus. The review focused on consistency with the predefined condition definitions and the stimulus set’s overall coherence.

All modifications to the stimuli were based on internally consistent visual principles (e.g., local contrast indices), and details regarding subsequent validation and *post hoc* analysis are provided below. These alterations underwent an iterative review to ensure distinct differentiation between conditions and prevent extreme or inconsistent manipulations across the stimulus set. The primary objective was to maintain the perceptual plausibility and visual coherence of the stimuli rather than enforce strict numerical thresholds. Consequently, the adopted procedure reflects a qualitative design approach with constraints.

To validate the neatness manipulation, we conducted a separate norming study involving 20 independent participants (mean age = 33.1 years, SD = 7; 12 women, 8 men). All participants were volunteers and did not take part in the main experimental study. Each participant rated the full set of 126 stimuli on a 7-point scale of visual neatness (1 = not neat at all, 7 = extremely neat), evaluating surface characteristics such as cleanliness, orderliness, absence of dust, stains, and visible damage. Inter-rater reliability was high (ICC = 0.704 for single measures, 0.979 for average measures; *p* < 0.001, two-way mixed model, consistency type), indicating consistent judgments across participants.

A repeated-measures ANOVA revealed a strong and statistically significant effect of condition, *F*_(2, 38)_ = 280.20, *p* < 0.001, partial *η*^2^ = 0.936. The ratings followed the expected pattern: Untidy < Neutral < Neat. Pairwise comparisons (Bonferroni corrected) confirmed that all conditions differed significantly from each other (*p* < 0.001). Crucially, the mean difference between Untidy and Neutral stimuli was 2.87 points, the difference between Neutral and Neat stimuli was 1.21 points, and the total spread between Untidy and Neat conditions reached 4.08 points. These values, combined with a large effect size (*η*^2^ = 0.936), demonstrate that the manipulation of surface neatness was effective and robustly perceived as intended (Supplementary material 2).

To evaluate the possibility of low-level visual confounds and to verify the consistency of stimulus manipulations across experimental conditions, we conducted a *post hoc* analysis of objective image properties for all 126 stimuli. For each image, we computed six metrics that are commonly used to control for low-level features: (1) edge density, calculated using the Sobel operator to estimate the proportion of edge pixels relative to total image area; (2) local edge density (mean and standard deviation), computed within a sliding window to quantify intra-object variation in edge presence; (3) image entropy, representing the distribution of grayscale intensity values and used as a proxy for informational complexity; (4) mean luminance, measuring the average pixel brightness; and (5) luminance standard deviation, indexing the variability of brightness within the image. All metrics were calculated on grayscale-converted images using custom designed Python scripts (see Supplementary materials). Each measure was subjected to a one-way ANOVA with surface condition (neat, neutral, untidy) as the independent variable. No statistically significant differences were found across conditions in edge density (*F*_(2,123)_ = 1.46, *p* = 0.2), image entropy (*F*_(2,123)_ = 0.03, *p* = 1), mean luminance (*F*_(2,123)_ = 2.00, *p* = 0.14), local edge density mean (*F*_(2,123)_ = 1.62, *p* = 0.2), or local edge density standard deviation (*F*_(2,123)_ = 1.17, *p* = 0.3). Luminance standard deviation showed a trend toward significance (*F*_(2,123)_ = 2.96, *p* = 0.06) but did not exceed the conventional threshold. These results indicate that the neatness manipulations did not systematically co-vary with basic image features, reducing the likelihood of perceptual complexity or brightness as confounding factors.

### Experimental procedure

**Figure 2:**
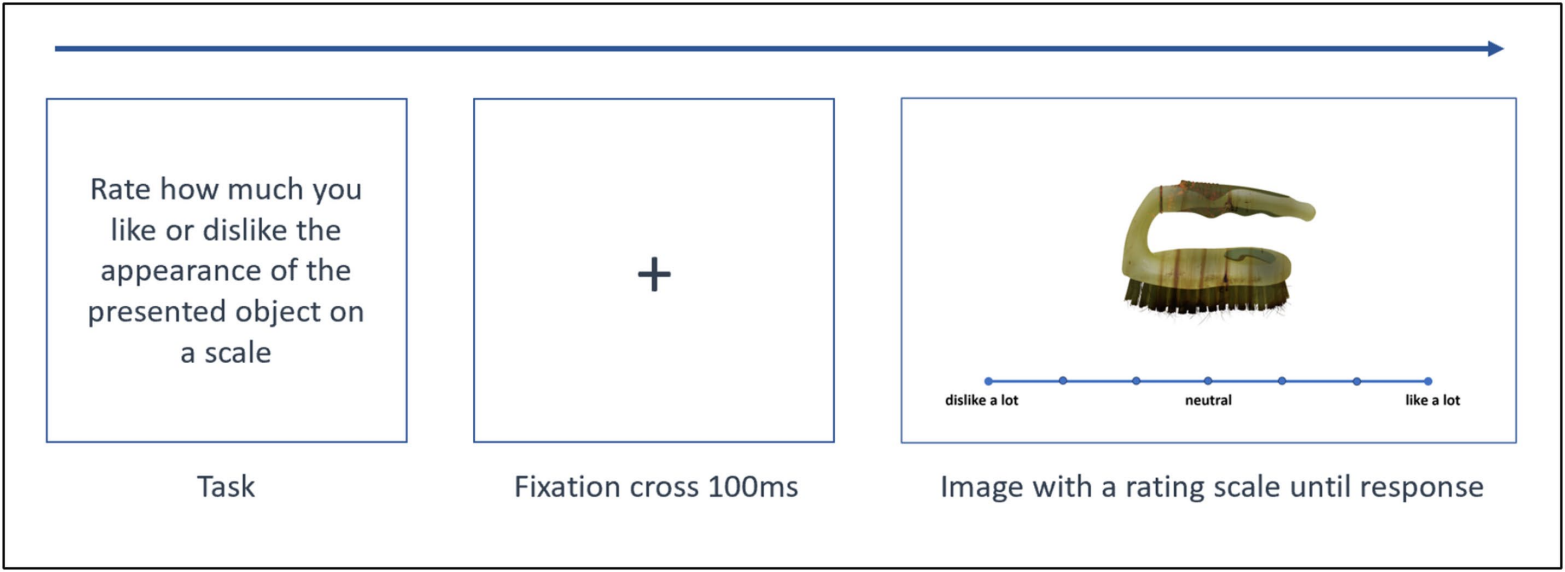
Experimental set-up and stimulus presentation.

Recordings took place in a sound-proof behavioral laboratory that had dim lighting and was equipped with an experimental set-up consisting of a 27-inch widescreen LCD monitor with a resolution of 1,920 × 1,080, a mouse, and a computer. The experiment was designed and presented to the participants using PsychoPy v.2022.2.4 software package. Participants were seated approximately 45 cm from the monitor. The following experimental instructions were given to participants: “Please rate on the scale how much you like or dislike the appearance of this object.” Participants were instructed to select one point on a 7-point Likert scale. The first, fourth and seventh points were labeled as “dislike a lot”, “neutral” and “like a lot”, respectively, with points in between marked with tickmarks only; no actual digits were present on the scale given, but responses were later transcoded to numerical scores from 1 (dislike a lot) to 7 (like a lot) programmatically for offline statistical evaluation (Figure 2).

### Data analysis

The dependent variable was the visual preference rating. A repeated-measures analysis of covariance (ANCOVA) was performed to analyze the main effects of and interactions between object neatness and object category, with MOCI scores included as a covariate to control for obsessive-compulsive tendencies. The Huynh-Feldt adjustments were applied when sphericity was violated (Blanca et al., 2023). In cases where statistically significant differences were identified, post-hoc contrasts were conducted with corrections applied according to the Bonferroni method. The effect sizes of the ANCOVA were calculated as partial eta squared values (*η*^2^), which were classified as small (0.01 ≤ *η*^2^ < 0.06), moderate (0.06 ≤ *η*^2^ < 0.14), or large (*η*^2^ ≥ 0.14).

In addition to the primary statistical analyses, we evaluated the inter-rater reliability of attractiveness judgments across participants using the intraclass correlation coefficient (ICC). Since each stimulus received a single-item rating from multiple independent raters, we applied a two-way mixed-effects model with a consistency definition (Shrout and Fleiss, 1979). This approach enabled us to evaluate the consistency of the evaluation patterns across all stimuli, providing a robustness check for the subjective ratings used as the dependent variable.

The raw data supporting the findings of this study are available at the OSF repository upon request.

## Results

All participants successfully completed data collection session and their data were submitted to ANCOVA to evaluate the effect of surface neatness and object category on visual preference rating. Table 1 presents the means and standard deviations for these data.

**Table 1:** Mean visual preference rating and standard deviations for different object categories across three surface states.

Object category	Overall		Surface state					
			Untidy		Neutral		Neat	
	Mean	SD	Mean	SD	Mean	SD	Mean	SD
Household items	3.71	0.66	1.72	0.53	4.40	0.74	5.02	0.72
Kitchen utensils	3.58	0.76	1.48	0.60	4.41	0.68	4.86	0.99
Personal items	3.56	0.68	1.40	0.49	3.95	0.77	5.33	0.78
Stationery	4.06*	0.65	1.99	0.66	4.68	0.76	5.52	0.53
Tools	3.68	0.83	1.72	0.66	4.25	0.92	5.10	0.91
Overall	3.72	0.72	1.66**	0.59	4.34**	0.77	5.17**	0.79

*The mean difference between stationery and other categories is significant at 0.05 level, with Bonferroni adjustment.

**The mean difference between all three surface states is significant at 0.05 level, with Bonferroni adjustment.

Mauchly’s test indicated that the assumption of sphericity was violated (for surface: *χ*^2^(2) = 13.43, *p* < 0.05; for Category: *χ*^2^(9) = 24.35, *p* < 0.05; for Surface-Category interaction: *χ*^2^(35) = 93.21, *p* < 0.05). Consequently, degrees of freedom were corrected using Huynh-Feldt estimates of sphericity (*ε* > 0.6) (Blanca et al., 2023).

**Figure 3:**
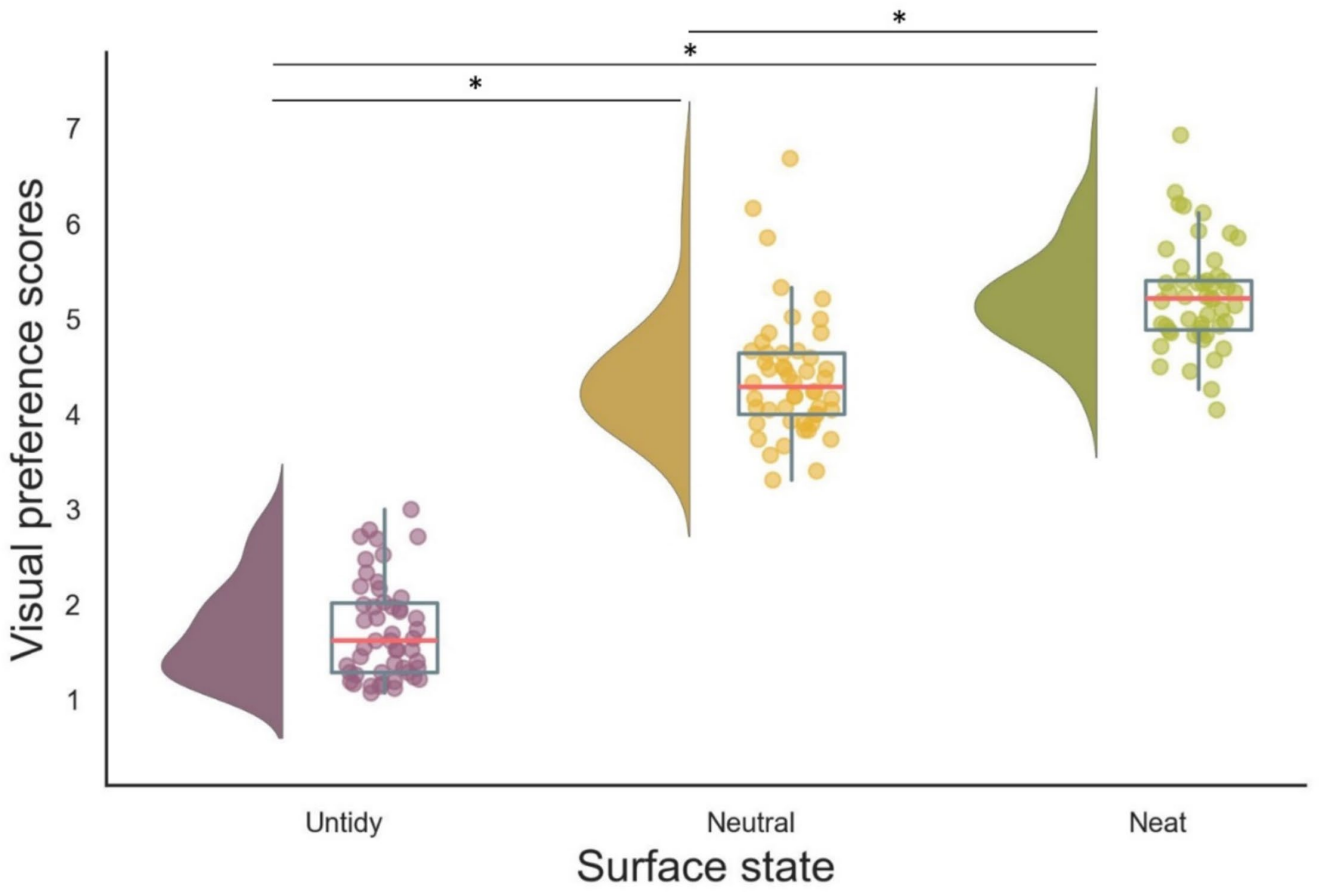
Raincloud plots of mean visual preference scores across surface neatness states. Mean visual preference rating for each object is shown as a colored dot. The median for each surface state is represented by a red line, the interquartile range (IQR) is depicted by the box, and whiskers indicate the 95% confidence interval (CI). The violin shapes illustrate the smoothed distribution of ratings for each neatness condition. *p < 0.05, Bonferroni-adjusted.

A significant main effect of surface neatness on visual preference scores was observed (*F*_(1.68, 80.79)_ = 89.15, *p* < 0.001, partial *η*^2^ = 0.65). This indicates the presence of a robust correspondence between surface neatness and visual preference. Namely, mean visual preference rating (see Table 1 and Figure 3) for objects with untidy surfaces were significantly lower than those for objects with neutral surfaces (mean difference = 2.68, *p* < 0.05), whereas the mean visual preference rating for objects with neatly presented surfaces were even higher than those for objects with neutral surfaces (mean difference = 0.83, *p* < 0.001).

**Figure 4:**
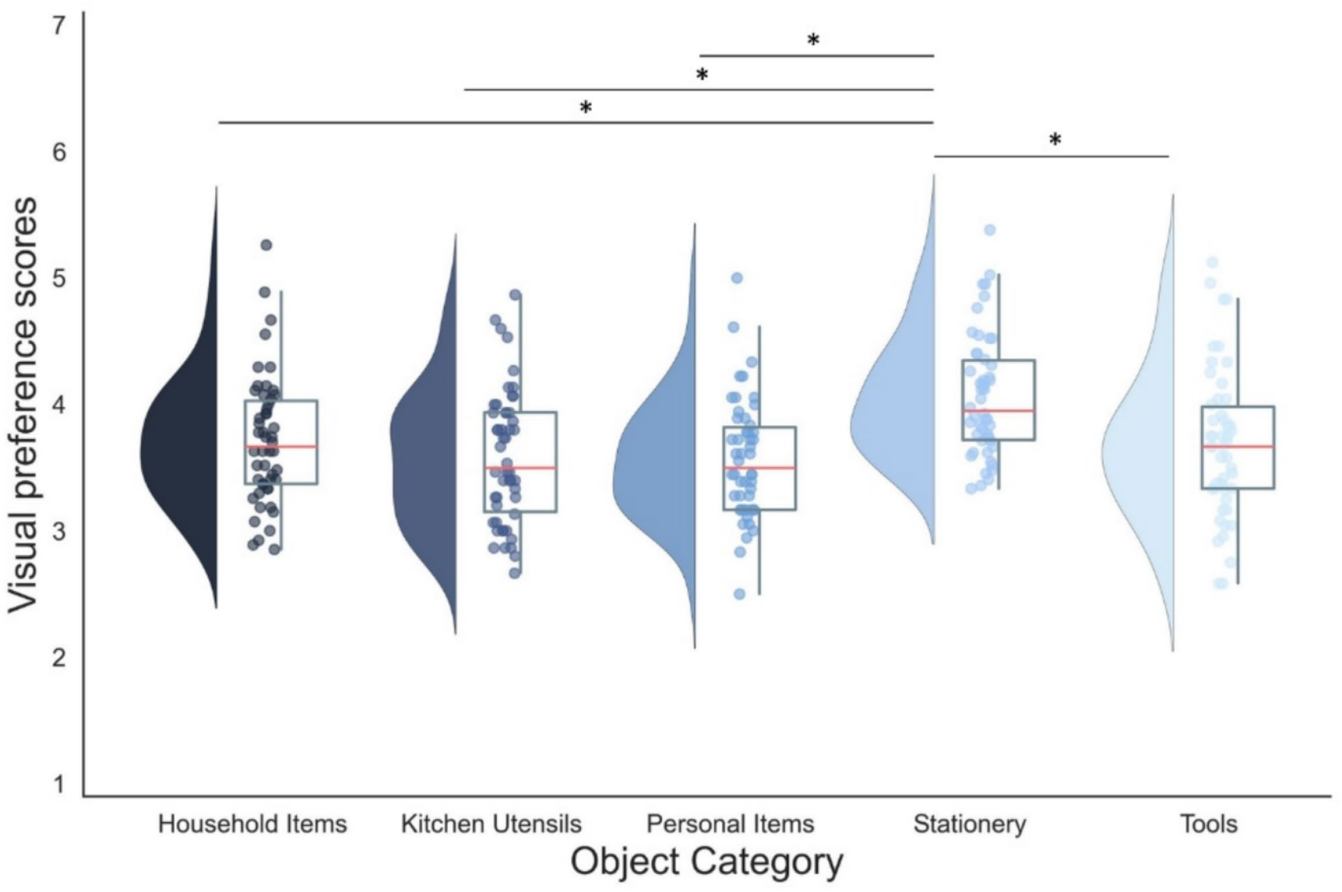
Raincloud plots of mean visual preference scores across categories. The distribution of visual preference scores for each category are shown as half-violin plots of different shades. The median for each category is represented by a red line, while the interquartile range (IQR) is depicted by the box. The whiskers extend to the 95% confidence intervals (CI) of the data. *p < 0.05 with Bonferroni adjustment.

Additionally, a significant main effect of category was observed (*F*_(3.24, 171.64)_ = 3.19, *p* < 0.05, partial *η*^2^ = 0.062). Subsequent post-hoc comparisons demonstrated that this was due to the stationery category receiving higher visual preference rating than all other categories (*p* < 0.05) (see Figure 4). No significant interactions between the OCD score and the factors of interest were identified (*p* > 0.05).

The Intraclass Correlation Coefficient (ICC) for visual preference ratings indicated moderate-to-high consistency among participants. Specifically, the ICC for single measures was 0.667 (95% CI: 0.612–0.724), suggesting acceptable agreement at the individual level. The ICC for average measures was 0.990 (95% CI, 0.987–0.992), *F*_(125, 6,125)_ = 101.27, *p* < 0.001, reflecting excellent reliability of the aggregated ratings.

## Discussion

The primary objective of this study was to systematically examine how surface neatness influences visual preference across various categories of everyday objects. In line with the established practice in empirical aesthetics, we interpreted these preference ratings as a proxy for perceived aesthetic value (Leder and Nadal, 2014; Palmer et al., 2012; Vessel et al., 2014). By manipulating surface conditions (from untidy to neutral to neat) we aimed to investigate how changes in surface neatness affect participants’ visual preference ratings, operationalized as like/dislike responses.

First, our study revealed a clear negative impact of untidiness on perceived attractiveness. Objects with hygienic or mechanical imperfections on their surface (obvious signs of use, e.g., stains, scratches, scuffs, greasiness, etc.) were rated as significantly less attractive, as compared with those in the neutral state (means scores 1.66 vs. 4.34, on a 1–7 scale). This aligns with the notion that mechanical or hygienic defects detract from an object’s visual appeal. Previous research has shown that dirt and related imperfections are generally perceived as aesthetically negative and trigger avoidance behaviors (Curtis et al., 2011; Karana et al., 2016; Mugge et al., 2018; Shook et al., 2019; White et al., 2016). In our study, the untidy features may have likely evoked similar feelings of aversion, leading to lower preference ratings, interpreted here as reduced visual appeal.

Second, cleanliness and neatness cues, such as shine, gloss, or simple geometric patterns, positively influenced the perceived attractiveness indicating that the objects with neat surfaces were rated as more attractive than neutral ones (mean = 5.17 vs. 4.34). These results are consistent with previous studies (Chadwick and Kentridge, 2015; Leddy, 1997; Sharma and Kumar, 2023; Silvia et al., 2018; Zhong et al., 2010). However, unlike the substantial drop observed for untidy surfaces (mean = 2.68), the attractiveness ratings for neat surfaces were more proximal to neutral ones. This asymmetry may be linked to the negativity bias, which is the well-documented sensitivity to negative stimuli, such as dirt or signs of contamination (Larsen and Cacioppo, 1998; Lazarus, 2021; Rozin and Royzman, 2001). Taken together, our results support a continuum-based interpretation of surface neatness, where the removal of negative cues and the addition of positive ones both contribute to the perception of surface quality and modulate visual preference.

The observed inter-rater reliability for single visual preference ratings (ICC = 0.667) is in the moderate range. This is consistent with the known variability in aesthetic evaluations across individuals. Previous studies have shown that people often have different preferences regarding what features they find attractive. For instance, Jacobsen (2004) demonstrated that participants evaluating the same abstract images relied on different visual features when judging beauty. Similarly, Leder and Nadal (2014) model of aesthetic experience suggests that aesthetic responses depend on a combination of perceptual and emotional assessments, which can differ across individuals. In this context, moderate agreement between participants is not surprising. At the same time, the very high reliability of averaged ratings (ICC = 0.990) confirms that effects at the condition level were consistent and interpretable at the group level.

In addition to the significant influence of surface neatness, our study revealed a main effect of object category on visual preference ratings. In particular, stationery items consistently received higher visual preference ratings regardless of surface neatness. The category effect aligns with prior research demonstrating that the perceived appeal of visual features, such as color, texture, symmetry, and gloss, is modulated by object category. For instance, studies have shown that color preferences are contingent on the object to which a color is applied (Holmes and Buchanan, 1984; Schloss et al., 2013). Palmer and Schloss proposed that preferences for colors reflect the average affective valence of objects typically associated with those colors, a principle formalized in their ecological valence theory (Palmer and Schloss, 2010). While this account specifically addresses color, it supports the broader notion that semantic associations linked to object categories can shape aesthetic responses. The present findings suggest that surface neatness may also be subject to such modulation, thereby extending the scope of category-dependent aesthetic effects to a novel and relatively understudied property.

The higher ratings assigned to stationery items may be putatively explained by the following factors. One possibility is related to exposure: participants (students mostly) may interact more frequently with stationery in their daily routines, leading to more positive affective responses through mere exposure (Bornstein, 1989). However, given the absence of an assessment of actual usage frequency in this study, this interpretation remains speculative; future research may address this question via a more direct examination of the role of exposure. Another explanation may be found in the symbolic and functional associations of object categories. Stationery items, including (but not limited to) pens, erasers, highlighters have been demonstrated to be associated with cognitive activity, planning, and productivity. These associations stand in contrast to those of other categories: household items (e.g., a brush) relating to domestic maintenance; kitchen utensils (e.g., a ladle)—to food preparation; personal-use objects (e.g., a comb)—to hygiene routines; and tools (e.g., a hammer)—to manual labor. A considerable number of these categories entail physical exertion or routine maintenance, i.e., domains that may bear a diminished affective valence in the context of aesthetic evaluation.

Conversely, stationery objects may benefit from more positive associations. Beyond their utilitarian function, these objects frequently serve as symbols of competence, order, and intellectual identity, particularly in academic or professional settings (Tian and Belk, 2005). These symbolic and semantic resonances have the potential to enhance the aesthetic appeal of stationery. Despite the absence of direct measurement of participants’ subjective associations in our study, the findings are consistent with the notion that judgments of surface aesthetics are influenced, at least in part, by the semantic framing and symbolic value of the object. This perspective is corroborated by extant research demonstrating that preferences are influenced not only by perceptual attributes but also by perceived appropriateness, typicality, and affective associations within an object’s conceptual context (Schloss et al., 2013). Clearly, the putative explanations of this finding are not mutually exclusive and are still in need of further research and validation.

### Limitations and future directions

Although the present study provides compelling evidence for the influence of surface neatness on perceived object attractiveness, several limitations and avenues for future research require further consideration.

First, although the present study clearly distinguished between untidy, neutral, and neat surface states, it is important to recognize that the concept of neatness is not a binary state, but rather a continuum. The objective of the study was not to ascertain the precise quantity of visual cues that elicit a perception of neatness or untidiness. However, it seems probable that this threshold varies from one individual to another, as the concept of neatness is culturally and socially determined. It would be beneficial for future research to investigate the relationship between surface neatness and aesthetic perception in a variety of cultural and linguistic contexts.

Second, it is possible that the relatively reduced effect for neat vs. neutral objects and the significant differences in attractiveness across object categories may be attributed to some limitations in the stimulus creation process. Despite the involvement of professional designers and the stringent validation procedures, the techniques used to manipulate neatness are still not fully standardized. Therefore, future research should develop a more systematic framework for classifying and quantifying neatness and untidiness cues, perhaps using standardized scales or computer vision algorithms.

Another potential limitation is the lack of explicit parametrization of surface modifications in terms of pixel-level metrics or perceptual gloss indices. While all stimuli were edited under tightly controlled visual guidelines and reviewed for consistency by an expert, and while we confirmed across six objective image metrics that low-level confounds were minimized, the manipulations were based on qualitative criteria rather than quantitative thresholds. We therefore cannot precisely quantify the extent to which visual attributes such as gloss, smudge distribution, or stain contrast varied across conditions. Future research could consider supplementing expert-controlled editing protocols with computational models to more formally calibrate visual manipulations across experimental stimuli.

Moreover, it is worth mentioning that this study focuses on the perception of detached objects, presented in isolation and devoid of the context in which they are typically encountered. While this approach does not provide a comprehensive understanding of object perception during real-world interactions—where both context and dynamic engagement are critical—it serves as a reasonable starting point for one of the first studies on this topic. This approach allows us to establish controlled conditions for studying the impact of surface characteristics on object perception. The use of detached objects is widely practiced in research on cognitive processes such as memory, visual search, and object recognition. Furthermore, such an approach is used in assessing cognitive functions, neuropsychological testing, and the development of rehabilitation programs. As research progresses, this work can be extended to incorporate more complex contexts, including physical interactions with objects as well as their social and linguistic dimensions.

Finally, the present study’s reliance on a uniform population sample (university students) limits the generalizability of the results to other populations. We also acknowledge that the perception of neatness varies substantially depending on cultural norms, individual differences, personal experiences, and professional expertise, particularly in fields involving manual labor. Consequently, future studies should replicate and extend the present findings with a more diverse participant pool to assess the universality of the observed effects.

## Conclusion

Our findings demonstrate that surface neatness significantly influences the perceived aesthetic appeal of everyday objects. Untidiness was consistently found to diminish attractiveness, while neatness enhanced it. These results have profound implications for research on human-object interactions, informing investigations into how the appearance of everyday objects affects cognition, emotions, behavior, and decision-making. Furthermore, our findings have direct practical applications in fields such as design, marketing, and consumer psychology. Designers can leverage these insights to create more appealing and desirable products, while marketers can employ strategies to emphasize the neatness of their offerings. Moreover, by highlighting the role of surface neatness in shaping aesthetic value, our data contribute to a growing body of research that seeks to prolong the lifespan of products and reduce consumption, thereby offering valuable perspectives on sustainability.

## Data Availability

The datasets presented in this study can be found in online repositories. The names of the repository/repositories and accession number(s) can be found below: OSF repository: https://osf.io/356rs/?view_only=0914891f606044e4984b5a4f82d1be64.
