# Supplementary material for: Surface neatness as an index of aesthetic value of everyday objects
Source: Front Psychol. 2025 Jul 10;16:1578785. doi: 10.3389/fpsyg.2025.1578785 (PMC12288685; doi:10.3389/fpsyg.2025.1578785)
Supplement: Supplementary file 1 [file Table_1.docx]

**Supplementary Table**1. Results of the manipulation check on perceived neatness.

Twenty independent participants rated each of 126 stimuli on a 7-point scale (1 = not neat at all, 7 = very neat). Below are the descriptive statistics for each condition, as well as the results of the repeated-measures ANOVA and Bonferroni-corrected pairwise comparisons.

| Condition | M (SD) | Pairwise Comparison | Mean Diff | SE | p-value* | 95% CI | Cohen’s *d* |
| --- | --- | --- | --- | --- | --- | --- | --- |
| Neat | 5.68 (0.82) | Neat vs Neutral | 1.21 | 0.12 | < 0.001 | [0.89, 1.53] | 1.67 |
| Neutral | 4.47 (0.68) | Neat vs Untidy | 4.08 | 0.23 | < 0.001 | [3.48, 4.69] | 5.56 |
| Untidy | 1.60 (0.61) | Neutral vs Untidy | 2.87 | 0.16 | < 0.001 | [2.45, 3.29] | 4.81 |

*all p-values are Bonferroni-corrected.

ANOVA (within-subjects): *F*_(2, 38)_ = 280.20, *p* < .001, partial η² = 0.936, observed power = 1.000
