# Supplementary material for: Surface neatness as an index of aesthetic value of everyday objects
Source: Front Psychol. 2025 Jul 10;16:1578785. doi: 10.3389/fpsyg.2025.1578785 (PMC12288685; doi:10.3389/fpsyg.2025.1578785)
Supplement: Supplementary file 2 [file Supplementary_file_1.docx]

**Stimulus Design and Manipulation Protocol**

This appendix outlines the technical specifications and design guidelines provided to the professional designer for the creation of visual stimuli used in the study on surface neatness perception.

A.1. General Requirements

The stimulus set comprised 42 original everyday objects sourced from the Bank of Standardized Stimuli (BOSS) database. For each original object, two modified versions were created: a 'neat' version and an 'untidy' version, resulting in a total of 126 images.

- File Format: All images were delivered in PNG format.
- Dimensions: Each image had dimensions of 2000 × 2000 pixels.
- Color Depth: Images maintained a 32-bit color depth.
- Alignment and Scaling: All objects were precisely centered on a square canvas and scaled proportionally to reflect their approximate real-world physical dimensions.

A.2. Surface Condition Specifications

A.2.1. Neat Condition

The objective for the 'neat' condition was to visually convey an impression of cleanliness, well-maintained surface, and overall tidiness. Permissible visual interventions included:

- Gloss and Highlights: addition of realistic highlights or gloss effects using soft, luminous gradients to simulate light reflection on a polished surface. The size and shape of these highlights were required to conform naturally to the object's form.
- Decorative Patterns (Optional): in cases where the original object possessed low intrinsic visual complexity, simple, neutral geometric patterns were permitted. These patterns, such as subtle dot grids, thin diagonal hatching, or low-contrast stripes, were strictly forbidden from containing symbols, overt decorative motifs, or cultural references.
- Constraints:
  - The fundamental shape of the object was not to be altered.
  - Unnatural or unrealistic color changes were prohibited.
  - Stylization resembling commercial advertising imagery was to be avoided.

A.2.2. Untidy Condition

The objective for the 'untidy' condition was to visually communicate a state of contamination, wear, or traces of use. Each untidy image was required to incorporate at least two distinct types of imperfections from the following list:

- Scratches: Rendered as fine, linear disruptions with irregular, eroded edges.
- Smudges/Grease Marks: Applied as semi-transparent, hazy, or opaque overlays.
- Fingerprints: Subtle, blurred, or indistinct impressions.
- Dust, Scuffs, and Contaminated Areas: Inclusion of general particulate matter, surface abrasions, or visibly soiled regions.
- Technical Parameters for Imperfections:
  - All defects were to be applied on separate, editable layers.
  - The scale of defects was to be consistent with the object's real-world dimensions.
  - The transparency of defects was to range between 30% and 70%.
- Critical Requirement: for objects with high intrinsic detail, moderation in adding imperfections was crucial to prevent visual overload.

A.3. General Image Manipulation Guidelines

- The fundamental form and orientation of all objects were to remain unaltered across all conditions.
- All image manipulations were to be exclusively performed using Adobe Photoshop 2022.
- All defects, patterns, and enhancements were to be applied on separate, non-destructive, and editable layers.
- The use of symbols, letters, logos, or illustrative elements (beyond the specified patterns) was strictly prohibited.

A.4. Quality Control and Standardization

A rigorous quality control process was implemented to ensure the consistency and perceptual validity of the stimulus set. Each object, across its three versions (neat, neutral, untidy), was systematically compared against the following parameters:

- Form and Scale Identity: Verification that the object's form and scale remained identical across all three conditions.
- Neatness Differentiation: Confirmation of clear and discernible visual differences in the level of neatness among the three conditions.

All modified images underwent a two-stage review process, conducted by both the project's art director and the scientific supervisor. Images failing to meet the specified criteria were returned for revision with detailed feedback.
